# Macrophage Stimulating Protein Enhances Hepatic Inflammation in a NASH Model

**DOI:** 10.1371/journal.pone.0163843

**Published:** 2016-09-29

**Authors:** Jieyi Li, Dipanjan Chanda, Patrick J. van Gorp, Mike L. J. Jeurissen, Tom Houben, Sofie M. A. Walenbergh, Jacques Debets, Yvonne Oligschlaeger, Marion J. J. Gijbels, Dietbert Neumann, Ronit Shiri-Sverdlov

**Affiliations:** 1 Department of Molecular Genetics, NUTRIM School of Nutrition and Translational Research in Metabolism, Maastricht University, Maastricht, The Netherlands; 2 Department of Molecular Genetics, CARIM School for Cardiovascular Diseases, Maastricht University, Maastricht, The Netherlands; 3 Department of Pharmacology, Maastricht University, Maastricht, The Netherlands; 4 Department of Medical Biochemistry and Experimental Vascular Biology, Academic Medical Center, University of Amsterdam, Amsterdam, The Netherlands; INRA, FRANCE

## Abstract

Non-alcoholic steatohepatitis (NASH) is a common liver disease characterized by hepatic lipid accumulation (steatosis) and inflammation. Currently, therapeutic options are poor and the long-term burden to society is constantly increasing. Previously, macrophage stimulating protein (MSP)—a serum protein mainly secreted by liver—was shown to inhibit oxidized low-density lipoprotein (OxLDL)/lipopolysaccharides (LPS)-induced inflammation in mouse macrophages. Additionally, MSP could reduce palmitic acid (PA)-induced lipid accumulation and lipogenesis in the HepG2 cell line. Altogether, these data suggest MSP as a suppressor for metabolic inflammation. However, so far the potential of MSP to be used as a treatment for NASH was not investigated. We hypothesized that MSP reduces lipid accumulation and hepatic inflammation. To investigate the effects of MSP in the early stage of NASH, low-density lipoprotein receptor (*Ldlr*^*-/-*^) mice were fed either a regular chow or a high fat, high cholesterol (HFC) diet for 7 days. Recombinant MSP or saline (control) was administrated to the mice by utilizing subcutaneously-implanted osmotic mini-pumps for the last 4 days. As expected, mice fed an HFC diet showed increased plasma and hepatic lipid accumulation, as well as enhanced hepatic inflammation, compared with chow-fed controls. Upon MSP administration, the rise in cholesterol and triglyceride levels after an HFC diet remained unaltered. Surprisingly, while hepatic macrophage and neutrophil infiltration was similar between the groups, MSP-treated mice showed increased gene expression of pro-inflammatory and pro-apoptotic mediators in the liver, compared with saline-treated controls. Contrary to our expectations, MSP did not ameliorate NASH. Observed changes in inflammatory gene expression suggest that further research is needed to clarify the long-term effects of MSP.

## Introduction

Non-alcoholic fatty liver disease (NAFLD) is a metabolic disorder which comprises a wide spectrum of liver damage, ranging from simple steatosis to steatohepatitis, liver fibrosis and cirrhosis. Non-alcoholic steatohepatitis (NASH) represents the stage that is composed of steatosis and hepatic inflammation, and is regarded as the hepatic manifestation of the metabolic syndrome. Although steatosis is considered relatively benign, the presence of inflammation is detrimental, as it may cause irreversible liver damage and sets the stage for further liver injury, like cirrhosis and liver cancer [1]. Currently, the mechanisms that trigger inflammation are unknown. Consequently, therapeutic options of NASH are poor and the long-term burden to society is constantly increasing.

Macrophage stimulating protein (MSP) is a serum protein, which is mainly secreted by hepatocytes [2]. It exerts its biological effects through binding to the receptor tyrosine kinase Recepteur d'Origine Nantais (receptor tyrosine kinase RON)–a transmembrane receptor, which is expressed in epithelial organs, including liver [3]. Since its discovery, the MSP-RON signaling pathway has been documented as a suppressor of exogenous substances (*e*.*g*. lipopolysaccharide (LPS) or galactosamine-induced inflammation) in multiple tissues [4–6]. Additionally, evidences point towards a beneficial role of MSP in hepatic lipid and glucose metabolic regulation. Homozygous MSP knockout (MSP-/-) mice were found to develop hepatic steatosis, even when fed regular chow [7]. Furthermore, MSP administration led to inhibition of cAMP/dexamethasone-induced gluconeogenesis in primary hepatocytes of both human and rat [8]. Our previous study showed that MSP could inhibit palmitic acid (PA)- and LPS-induced upregulation of pro-inflammatory cytokines in mouse primary hepatocytes. MSP was also found to reduce PA-induced lipid accumulation and lipogenesis in the HepG2 cell line [9]. Moreover, when challenged with LPS and oxidized low-density lipoprotein (OxLDL), which can be considered a metabolic hazard for the development of NASH [10], the pro-inflammatory cytokine production was inhibited by MSP in mouse bone marrow-derived macrophages (BMDMs) [9]. These findings suggest that MSP acts as a negative regulator of lipid-induced inflammation *in vitro*. So far, the systemic effect of MSP in the context of the metabolic syndrome has not been investigated. In the current study, we investigated the role of MSP in a hyperlipidemic mouse model in order to determine its clinical potential in the field of NASH. We hypothesized that MSP leads to a reduction of fat accumulation and hepatic inflammation *in vivo*.

To test this hypothesis, hyperlipidemic low-density lipoprotein receptor knockout (*Ldlr*^*-/-*^) mice, fed a high fat, high cholesterol (HFC) diet for 1 week, were used as a mouse model for NASH. To elucidate the therapeutic effects of MSP, recombinant MSP was consecutively administered to mice with assistance of a subcutaneously-implanted osmotic mini-pump. We analyzed the changes in lipid accumulation, inflammatory cell infiltration, and relative gene expressions in the liver. Unexpectedly, we found that MSP promoted rather a pro-inflammatory, instead of anti-inflammatory, response as judged by relevant gene expression levels. Therefore, future studies are needed to evaluate the long-term effects of MSP to better understand its role in NASH.

## Materials and Methods

### Mice, diet and treatment

Mice were housed under standard conditions and given unlimited access to food and water. Experiments were performed according to Dutch regulations and approved by the Committee for Animal Welfare of Maastricht University. Female 10–12 week old *Ldlr*^*-/-*^ mice were placed on either chow or an HFC diet for 7 days. The HFC diet contained 17% casein, 0.3% DL-methionine, 34% sucrose, 14.5% cornstarch, 0.2% cholesterol, 5% cellulose, 7% CM 205B, 1% vit 200, 21% butter (diet code 1635; Scientific Animal Food and Engineering, Villemoissonsur-Orge, France). After 3 days, mice fed on each diet were administered with either recombinant MSP (500 ng/day, U-Protein Express BV, Utrecht, NL), or saline for 4 consecutive days (MSP chow: n = 8, HFC: n = 8; saline chow: n = 8; HFC: n = 8). Consecutive administration of recombinant MSP or saline was achieved by utilizing the osmotic mini-pumps (Alzet 2001, DURECT Corporation, Cupertino, CA, USA). Osmotic mini-pumps were placed subcutaneously in the back region of the mouse under isoflurane anesthesia. Blood was collected from the tail vein at the end of the experiment and mice were sacrificed afterwards. Liver tissue was harvested and snap-frozen in liquid nitrogen or fixed in 4% formaldehyde.

### Plasma/liver lipid measurements

Plasma cholesterol and triglycerides were measured via an enzymatic colorimetric assay according to the manufacturer’s protocol (Cholesterol Liquicolor CHOD_PAD; Human #10028, Instruchemie, Delfzijl) (Sigma Triglyceride (GPO Trinder) kit (Sigma Tr0100)). Absorbance was measured with the BioRad Benchmark Plate Reader (170-6750XTU; Bio-Rad, Hercules, CA). To measure liver cholesterol and triglycerides, liver homogenates were made. Approximately 40–50 mg of frozen liver tissue was homogenized in 1 ml SET buffer (250 mM Sucrose, 2 mM EDTA, 10 mMTris) with 1 mm glass beads (Biospec, art. 11079110) on the maximal setting of the Biospec Mini Bead Beater-1. Afterwards, samples underwent two freeze-thaw cycles for complete cell destruction. To optimize cell destruction, samples were taken through a 25Gx5/8” needle several times and a final thaw cycle was added. Total protein content was measured via bicinchoninic acid (BCA) assay (23225; Pierce, Rockford, IL). Liver cholesterol and triglycerides were measured via the enzymatic colorimetric assay.

### Liver histology

Frozen liver sections (7 μm) were fixed in acetone and subsequently blocked for endogenous peroxidase by incubation with 0.25% of 0.03% H_2_O_2_ for 5 minutes. Primary antibodies used were against infiltrated macrophages and neutrophils (rat-anti-mouse Mac-1 [M1/70]), and neutrophils (rat-anti-mouse Ly6-C, clone NIMP-R14) (generous gift from Prof Heeringa, Groningen, The Netherlands). 3-Amino-9 ethylcarbazole (AEC) (A85SK-4200.S1; Bio-connect, Huissen, The Netherlands) was applied as color substrate and hematoxylin (4085.9002, Klinipath, Duiven, The Netherlands) was used for nuclear counterstaining. TUNEL staining for apoptosis was performed on frozen liver sections according to the manufacturers' protocol (In situ Cell Death Detection Kit, Roche Applied Science). Sections were enclosed with Faramount aqueous mounting medium (S302580; DAKO, Glostrup, Denmark). For the lipid staining, the neutral lipid marker Oil Red O (ORO; O0625; Sigma-Aldrich) was used.

Paraffin-embedded liver sections (4 μm) were stained with Hematoxylin-Eosin (Eosin, E4382; Sigma-Aldrich). Images were taken with a Nikon digital camera DMX1200 and ACT-1 v2.63 software (Nikon Instruments Europe, Amstelveen, The Netherlands).

### RNA isolation and quantitative polymerase chain reaction

Total RNA was isolated from frozen mouse liver as described previously [11, 12]. First-strand complementary DNA (cDNA) was made from 500 ng total RNA of each mouse according to the manufacturer’s protocol (iScript™ cDNA Synthesis Kit (170–8891), Bio-Rad, Veenendaal, The Netherlands). Using 10 ng of cDNA template, relative quantitative gene expression levels were measured by quantitative PCR on an SDS 7900HT using SensiMix SYBR HIROX (Cat No QT605-05 Bioline, London, United Kingdom). Primers sets were developed with Primer Express version 2.0 (Applied Biosystems) using default settings. Data from qPCR were analyzed using the LinRegPCR software (Version 2015.3) [13–15].

### Western blotting

Approximately 40–50 mg of frozen liver tissue was homogenized in 1 ml RIPA (50 mM Tris-HCL pH 7.5, 150 mM NaCl, 0.5% Sodium deoxycholate, 1% Triton X-100, 0.1% SDS) supplemented with protease and phosphatase inhibitor mixture, with 1 mm glass beads on the maximal setting of the Biospec Mini Bead Beater-1. Equal amounts of protein (20 ug) were loaded onto the gel. After SDS/PAGE, proteins were transferred on nitrocellulose membrane (Bio-Rad). Subsequently, the membrane was blocked with 4% non-fat dry milk for 1 h at room temperature. For detection, the membrane was incubated with anti-bodies overnight at 4°C, followed by incubation with donkey anti-rabbit antibody for 1 h at room temperature. All antibodies were purchased from Cell Signaling Technology (Danvers, MA, USA). Signal was detected on autoradiograms by enhanced chemoluminescence.

### Measuring aminotranferases

The level of aminotransferases ALT in plasma of each individual mouse was measured using the Reflotron-system (Roche Diagnostics, Almere, The Netherlands), according to the manufacturer’s instructions.

### Statistical analysis

Data were analyzed using Graphpad Prism 6.01 (GraphPad Software, Inc., La Jolla, CA, USA). Groups were compared using two-way ANOVA. The data were expressed as the mean and standard error of the mean (SEM) and were considered significantly different at *p≤0.05; ** p<0.01; *** p<0.001.

## Results

### No difference in liver and spleen weights upon MSP treatment

**Fig 1:**
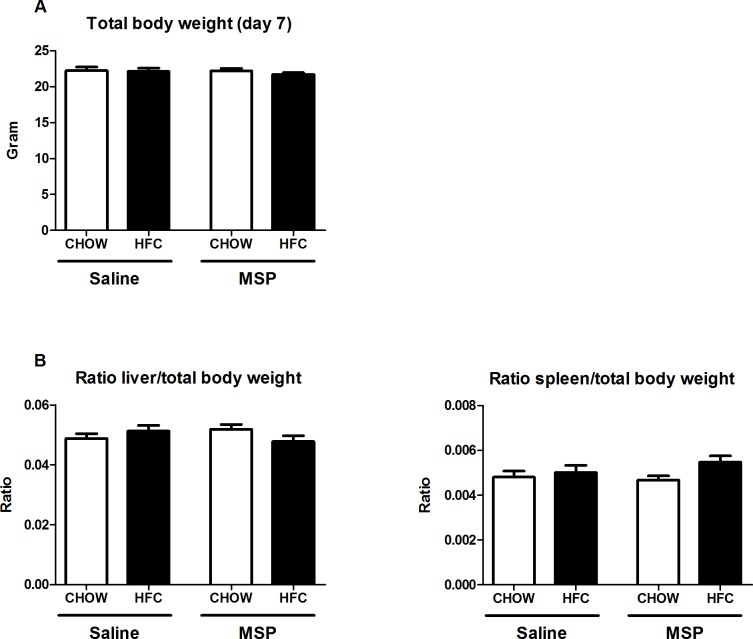
Relative liver and spleen weight. (A) Body weight after one week of regular chow or HFC diet in *Ldlr*^*-/-*^ mice with and without MSP treatment. (B) Relative liver and spleen weights after one week of regular chow or HFC diet in *Ldlr*^*-/-*^ mice with and without MSP treatment. Data are represented as mean ± SEM.

Compared to mice on regular chow diet, the total body weight did not change after one week of HFC diet and remained similar upon 4 days of MSP treatment (Fig 1A). Further, both mean liver and spleen weights were unaltered among the groups (Fig 1B), suggesting that neither one week of HFC diet, nor short-term MSP administration is sufficient to affect relative liver or spleen weight.

### Hepatic and plasma lipid accumulation upon MSP treatment remains similar

**Fig 2:**
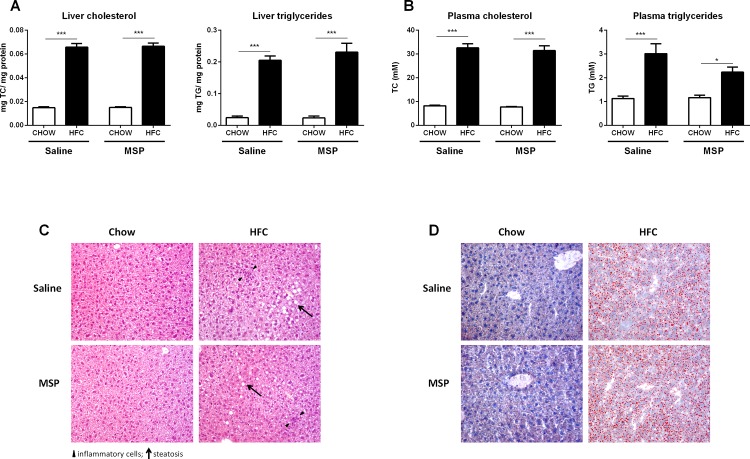
Liver and plasma lipid levels. (A) Hepatic levels of total cholesterol (TC) and total triglycerides (TG) after one week of regular chow or HFC diet in *Ldlr*^*-/-*^ mice, with and without MSP treatment. (B) Plasma TC and TG levels. (C,D) Representative images (200x magnification) of the H&E staining and Oil Red O staining of liver sections. Data are represented as mean ± SEM. * and *** indicate significant differences between groups, with * p≤0.05 and *** p<0.001.

As expected, compared with chow controls, the levels of total cholesterol (TC) and triglycerides (TG) were significantly elevated in both plasma and liver after one week of HFC diet. However, comparing saline- and MSP-treated mice on a HFC diet, no differences were found in hepatic TC and TG concentrations (Fig 2A). Similarly, plasma TC and TG levels did also not show differences between saline- and MSP-treated mice on a HFC diet (Fig 2B). In order to examine whether saline/MSP treatment and body weight (BW) were capable of predicting the plasma TG level in mice on HFC diet, a multiple linear regression was conducted. No significant regression equation was found (F(2, 13) = 1.392, p = 0.283), with an R^2^ of 0.176. Further, the analysis showed that treatment (Beta = -0.38, t = -1.49, ns) and BW (Beta = 0.11, t = 0.41, ns) did not significantly predict the value of plasma TG. In line with these data, HFC feeding resulted in equal levels of steatosis in the saline- and MSP- treated groups, as indicated by the H&E staining and Oil-red-O staining (Fig 2C and 2D), suggesting that short-term administration of MSP does not affect hepatic and plasma lipid concentration.

### No difference in hepatic macrophage and neutrophil infiltration upon MSP treatment

**Fig 3:**
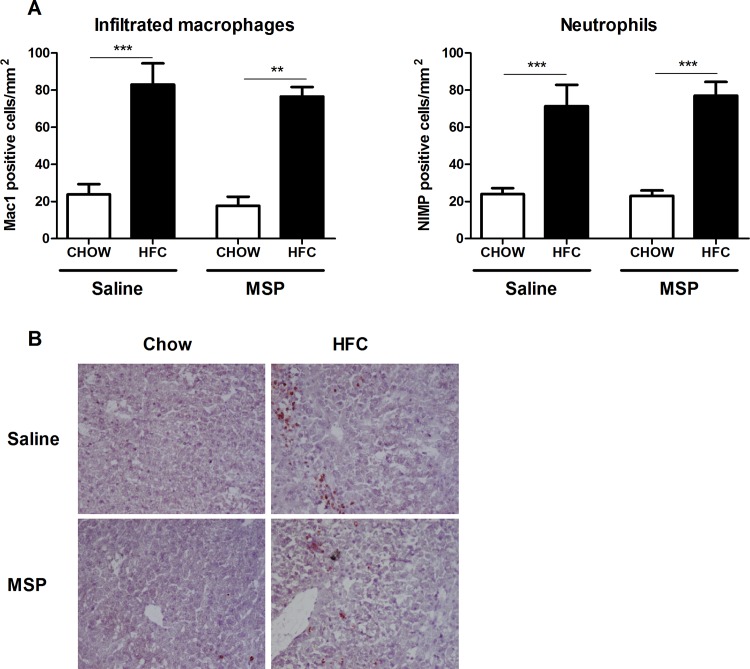
Parameters of macrophage and neutrophil infiltration in the liver. (A) Liver sections were stained for infiltrating macrophages and neutrophils, respectively. Positive immune cells were counted. (B) Representative images of the Mac1 staining (200× magnification) after one week of regular chow or HFC diet in *Ldlr*^*-/-*^ mice, with and without MSP treatment. Data are represented as mean ± SEM. ** and *** indicate significant differences between groups, with ** p<0.01 and *** p<0.001.

To determine the effect of MSP on hepatic inflammation in the context of NASH, liver sections were stained against Mac1 and NIMP, *i*.*e*. markers for infiltrating macrophages and neutrophils, respectively. As expected, scoring of stained sections revealed a higher amount of infiltrating macrophages and neutrophils in the liver of mice fed a HFC diet, compared with chow controls. However, no significant differences in macrophage and neutrophil infiltration were observed between saline- and MSP-treated mice on an HFC diet (Fig 3A and 3B). Clustering of macrophages in liver sections did not differ between saline- and MSP-treated mice (Fig 3C). Altogether, these data suggest that MSP treatment does not affect HFC-induced hepatic macrophage and neutrophil infiltration.

### Hepatic expression of pro-inflammatory and apoptotic genes is increased upon MSP treatment

**Fig 4:**
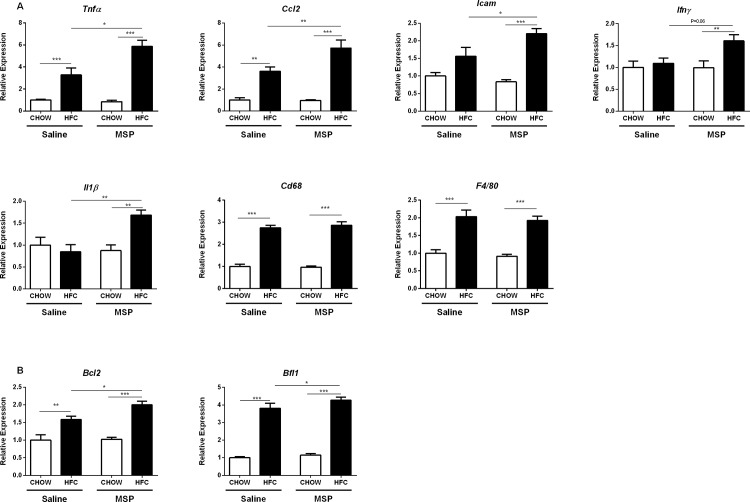
Hepatic expression levels of genes related to inflammation. (A) Gene expression levels of the pro-inflammatory cytokines, tumor necrosis factor alpha (*Tnfα*), chemokine (C-C motif) ligand 2 (*Ccl2*), intercellular adhesion molecule 1 (*Icam1*), interferon gamma (*Ifnγ*), interleukin 1 beta (*Il1β*), macrophage markers *F4/80* and Cluster of differentiation 68 (*Cd68*) in the livers of *Ldlr*^*-/-*^ mice on chow or HFC diet, with or without MSP treatment. (B) Hepatic gene expression levels of B-cell lymphoma 2 (*Bcl2*) and BCL2-related protein A1 (*Bfl1*). Data are represented as mean ± SEM. *, ** and *** indicate significant differences between groups, with * p≤0.05, ** p<0.01, *** p<0.001.

To further define the effects of MSP on early changes of hepatic inflammation, relative mRNA expression of pro-inflammatory genes in the liver were analyzed. The macrophage markers *F4/80* and Cluster of differentiation 68 (*Cd68*) were upregulated upon HFC diet, but remained similar in saline- and MSP- treated groups. Surprisingly, significantly higher, instead of lower, expression levels of several pro-inflammatory genes in the liver were observed in MSP-treated mice on an HFC diet, compared with controls. This was shown by an increased expression of the following genes: tumor necrosis factor alpha (*Tnfα*), chemokine (C-C motif) ligand 2 (*Ccl2*), intercellular adhesion molecule 1 (*Icam1*), interferon gamma (*Ifnγ*), and interleukin 1 beta (*IL1β*) (Fig 4A). Moreover, the apoptotic gene expression levels of B-cell lymphoma 2 (*Bcl2*) and BCL2-related protein A1 (*Bfl1*) were also increased upon MSP treatment in mice fed with a HFC diet (Fig 4B). Despite the fact that there were no histological changes observed in the livers of saline- and MSP-treated groups (Fig 3 and S4 Fig), the increase in hepatic mRNA levels of multiple inflammatory genes suggest the presence of early increased inflammation in the livers of MSP-treated *Ldlr*^*-/-*^ mice.

### Hepatic expression of fibrosis- and fatty acid metabolism-related genes is unaltered upon MSP treatment

**Fig 5:**
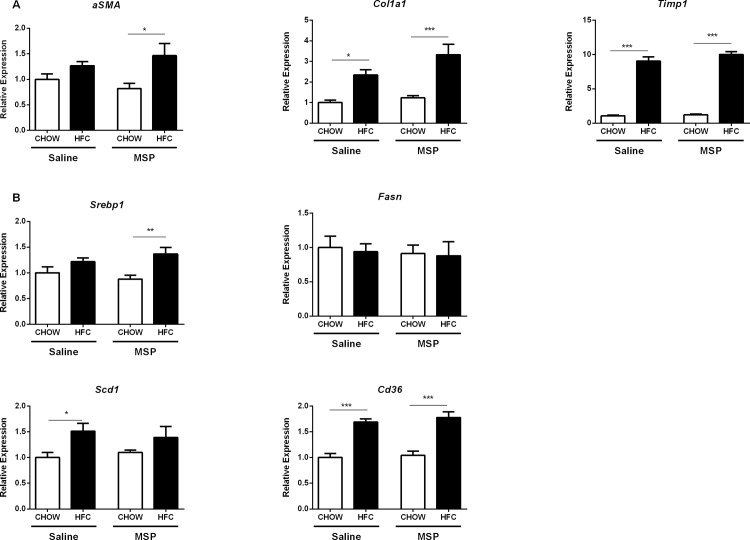
Hepatic expression levels of genes related to fibrosis and fatty acid metabolism. (A) Gene expression levels of the fibrotic makers alpha-smooth muscle actin (*αSMA*), collagen 1 type 1 (*Col1a1*) and TIMP metallopeptidase inhibitor 1 (*Timp1*) in the livers of *Ldlr*^*-/-*^ mice on chow or HFC diet, with or without MSP treatment. (B) Hepatic gene expression levels of Sterol regulatory element-binding transcription factor 1 (*Srebp1*), Stearoyl-CoA desaturase-1 (*Scd1*), Fatty acid synthase (*Fasn*), and Cluster of differentiation 36 (*Cd36*). Data are represented as mean ± SEM. *, ** and *** indicate significant differences between groups, with * p≤0.05, ** p<0.01, *** p<0.001.

To investigate the effects of MSP on fibrosis in the context of NASH, gene expression analysis of alpha-smooth muscle actin (*αSMA*), collagen 1 type 1 (*Col1a1*) and TIMP metallopeptidase inhibitor 1 (*Timp1*) was performed. Whereas the expression levels of these fibrotic genes were increased in the liver of HFC-fed mice, compared with chow-fed controls, no differences were found upon short-term treatment with MSP (Fig 5A), indicating that MSP does not affect the development of fibrosis. In order to investigate whether MSP regulates fatty acid metabolism in the liver, hepatic expression levels of Sterol regulatory element-binding transcription factor 1 (*Srebp1*), Stearoyl-CoA desaturase-1 (*Scd1*), Fatty acid synthase (*Fasn*), and Cluster of differentiation 36 (*Cd36*) were determined. While we observed an increasing trend of *Srebp1*, *Scd1*, *Fasn*, and *Cd36* in the HFC-fed group, no differences were observed between saline- and MSP- treated mice (Fig 5B), suggesting that liver fatty acid metabolism is not affected by MSP treatment.

## Discussion

In the present study, the role of MSP in lipid accumulation and hepatic inflammation was investigated for the first time in a mouse model for NASH. Our results showed that MSP could not ameliorate NASH in the early stage. Although data are inconclusive, the gene expression levels may suggest that MSP can promote the pro-inflammatory response in our model. These surprising results provide value to better comprehend the role of MSP in the metabolic syndrome.

MSP was first discovered in 1976 as a serum protein, which stimulates chemotactic responses, migration and spreading of peritoneal resident macrophages [16]. Subsequent evidences pointed out a correlation between MSP and liver injury; for example, MSP was transcriptionally up-regulated in the liver during hepatic inflammation and regeneration in rodent models [17]. Currently, the investigations of MSP in the context of hepatic inflammation are relatively limited, and are restricted to inflammation that is induced by exogenous substances [18]. Whereas MSP has emerged as a beneficial moderator in hepatic lipid and glucose metabolism [7, 8, 18], the role of MSP in the context of the metabolic syndrome, especially in metabolic inflammation, has not been explored. NASH, viewed as the hepatic manifestation of the metabolic syndrome, can be driven by the following risk factors: over-nutrition, lipid metabolites, production of pro-inflammatory cytokines and adipokines, gut bacteria and oxidation, among others. Cross-talk between metabolic organs, like adipose tissue and gut, may well participate in the pathogenesis of NASH [1, 19]. However, MSP does not confine its effects to the liver only; it has multiple roles in other organs as well, as the MSP receptor RON is expressed universally inside the body [3, 20]. Given the complexity of the pathogenesis and participation of organ cross-links, an *in vivo* NASH model will therefore offer more integrated insights in the systemic effects of MSP.

In the current study, we observed increased expression of several pro-inflammatory genes in the livers of mice affected with NASH. TNFα has been shown to be a key factor in the development of NAFLD and NASH [21, 22]. Since Kupffer cells, the liver’s residential macrophages, are the first responding cells to hepatocyte injuries, the increased release of TNFα from these cells is one of the most common characters of early phase of NASH in mouse model. Similarly, hepatic expression of *Ccl2* was also found elevated at an early stage in NASH mouse model, results that are consistent with data from NAFLD patients [23, 24]. Increases in Ccl2 expression lead to infiltration of pro-inflammatory monocytes and T cells, which induces the secretion of inflammatory cytokines such as TNFα and IL1β in the liver. ICAM1, which is involved in inflammatory cell migration and invasion, was also up-regulated in mice treated with MSP. In addition, IL1β, an inflammatory cytokine produced upon inflammasome activation, also plays an important role in NASH, as hepatocellular depletion of IL1β has been shown to protect mice from diet-induced steatohepatitis [25]. In the current study, our data showed increases in those pro-inflammatory gene expressions in response to MSP-treatment, when feeding mice a HFC diet, compared with saline-treated controls. This suggests a more severe inflammation after treatment of MSP, which is contrary to the mainstream point of view that MSP-RON acts as an anti-inflammatory mediator during an inflammatory reaction. Nevertheless, studies deliberating the role of MSP, particularly in liver inflammation, are contradictive and limited. On one hand, when compared with control mice, RON-/- mice (*i*.*e*. these mice have an abolished MSP-RON signaling pathway) exhibited reduced expression of the anti-inflammatory cytokine, interleukin 10 (IL10), and the anti-oxidant superoxide dismutase (SOD) in the liver, in response to LPS-induced acute endotoxemia [26]. On the other hand, loss of RON signaling led to an attenuated inflammatory response in the liver, when challenged with acetaminophen-induced hepatotoxicity [27]. Furthermore, conflict has also been tracked by Leonis *et al*., showing that RON Tyrosine Kinase (TK)-/- mouse (*i*.*e*. these mice are deficient for the TK domain of RON, in order to eliminate intracellular MSP-RON signaling) obtained increased serum pro-inflammatory cytokine levels, although their liver histology and liver damage was improved compared with control mice [28]. These results indicate that myeloid cells, compared to liver parenchyma cells, may react differently to MSP-RON signaling.

Although the reason for these contradictory findings is not clear, the hint that MSP-RON signaling exerts discriminating effects to different components within the liver fits the explanation. Kupffer cells and hepatocytes isolated from RON TK-/- were studied to further explore the specific effects of MSP on the liver. Compared with their wild type (RON TK+/+) controls, LPS-treated RON TK-/- Kupffer cells demonstrated more pronounced TNFα production, whereas RON TK-/- hepatocytes challenged with TNFα exhibited much better viability [4]. Moreover, mouse models with a specific deletion of RON in either the Kupffer cells or the hepatocytes were investigated and confirmed the diversity of MSP in those two different cell types. Compared with wild type control, hepatocyte-specific RON TK-/- mice exhibited lower alanine transaminase (ALT) levels and less apoptosis, accompanied with longer overall survival, while Kupffer cell-specific RON TK-/- mice showed the opposite pattern [4]. In short, disrupted MSP-RON signaling seems to evoke Kupffer cell activation and subsequent inflammatory cytokine production, but protects hepatocytes against a noxious stimulus. Therefore, in the current study, it is possible that the hepatocytes, in response to the MSP-RON activation, may have become more susceptible to the lipotoxic insult, which ultimately led to more severe cell damage and apoptosis, hence generating a pro-inflammatory hepatic phenotype.

Another explanation for the contradictive findings can be ascribed to the overall effect of MSP within the whole body, including cross-talk between various organs. Some evidence indicates that MSP can act as a pro-inflammatory mediator in other cells/ tissues. Recombinant MSP is found to promote the release of inflammatory factors and activate the nuclear factor kappa B (NFκB) pathway, a common inflammatory pathway, in isolated alveolar macrophages from both rat and patient [29, 30]. In addition, in a rodent model of mesangial proliferative nephritis, neutralization of MSP by means of anti-MSP antibodies attenuated inflammatory cell infiltration and eventually protected against further injury [31]. Moreover, adipose tissue from RON TK-/- mice fed an HFC diet showed less mass and significantly lower expression of TNFα in comparison to their wild type controls [32]. These data suggest that the effect of MSP on inflammation is complex; it participates in multiple pathological processes, which may bring variation in terms of ensemble effects.

It is noteworthy that, when investigating MSP-RON signaling, most previous studies were based on a disrupted RON signaling model. Yet, the current study utilized an enhanced MSP-RON signaling model, which certainly may bring some discrepancy. In the RON TK-/- model, the TK region of RON is deleted to block its downstream intracellular signaling. However, it is still possible that the RON TK-/- receptor interacts with other signal transduction pathways through receptor complexes. In fact, the hepatocyte growth factor (HGF) and its receptor c-Met, a close family member of MSP and RON in both structure and function [33–36], have been found to cooperate with the insulin receptor (INSR) by forming a c-Met-INSR hybrid complex, which puts HGF-c-Met in participation of insulin responsiveness [37]. It is therefore possible that enhanced MSP-RON signaling activates other signaling pathways, which could potentially interfere with MSP-RON-induced inflammatory responses. Nevertheless, no evidence has shown that MSP shares the same effects with HGF in insulin signaling transduction. In the current study, *Ldlr*^*-/-*^ mice fed a HFC diet for one week did not induce notable insulin resistance (IR) (S3 Fig). This result is in line with previous work, indicating that increased fasted insulin levels and HOMA-IR were only observed after 15 weeks of HFC diet in these mice [38, 39]. Given that, an advanced stage NASH model would be more suitable to investigate the effects of MSP on IR.

Overall, our data provide evidence for the first time that MSP could not ameliorate NASH in the hyperlipidemic *Ldlr*^*-/-*^ mouse model. Inversely, MSP could promote the expression of pro-inflammatory genes in the early stage of NASH. Therefore, future studies should evaluate the long-term effects of MSP in NASH.

## Supporting Information

S1 Fig Plasma ALT level. (TIF)

S2 Fig Scoring of steatosis and inflammation from histology. (TIF)

S3 Fig protein level of pAKT in liver. (TIF)

S4 Fig TUNEL staining and cell regeneration marker. (TIF)
