# Supplementary figures and images for: Macrophage Stimulating Protein Enhances Hepatic Inflammation in a NASH Model
Source: PLoS One. 2016 Sep 29;11(9):e0163843. doi: 10.1371/journal.pone.0163843 (PMC5042385; doi:10.1371/journal.pone.0163843)

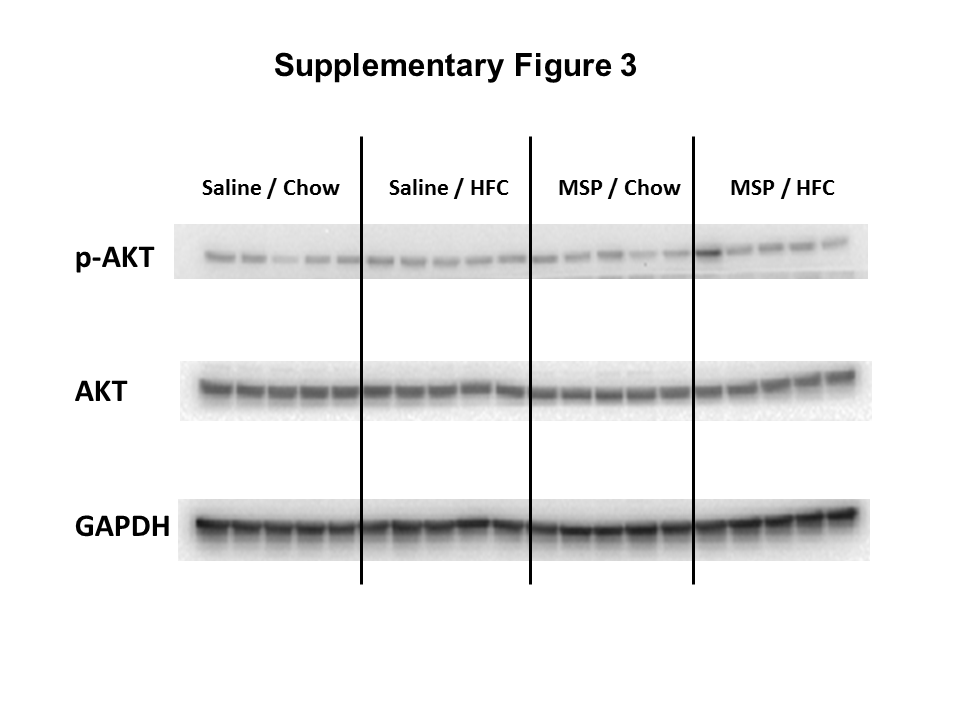

Supplement: S3 Fig — (TIF) [file pone.0163843.s003.tif]

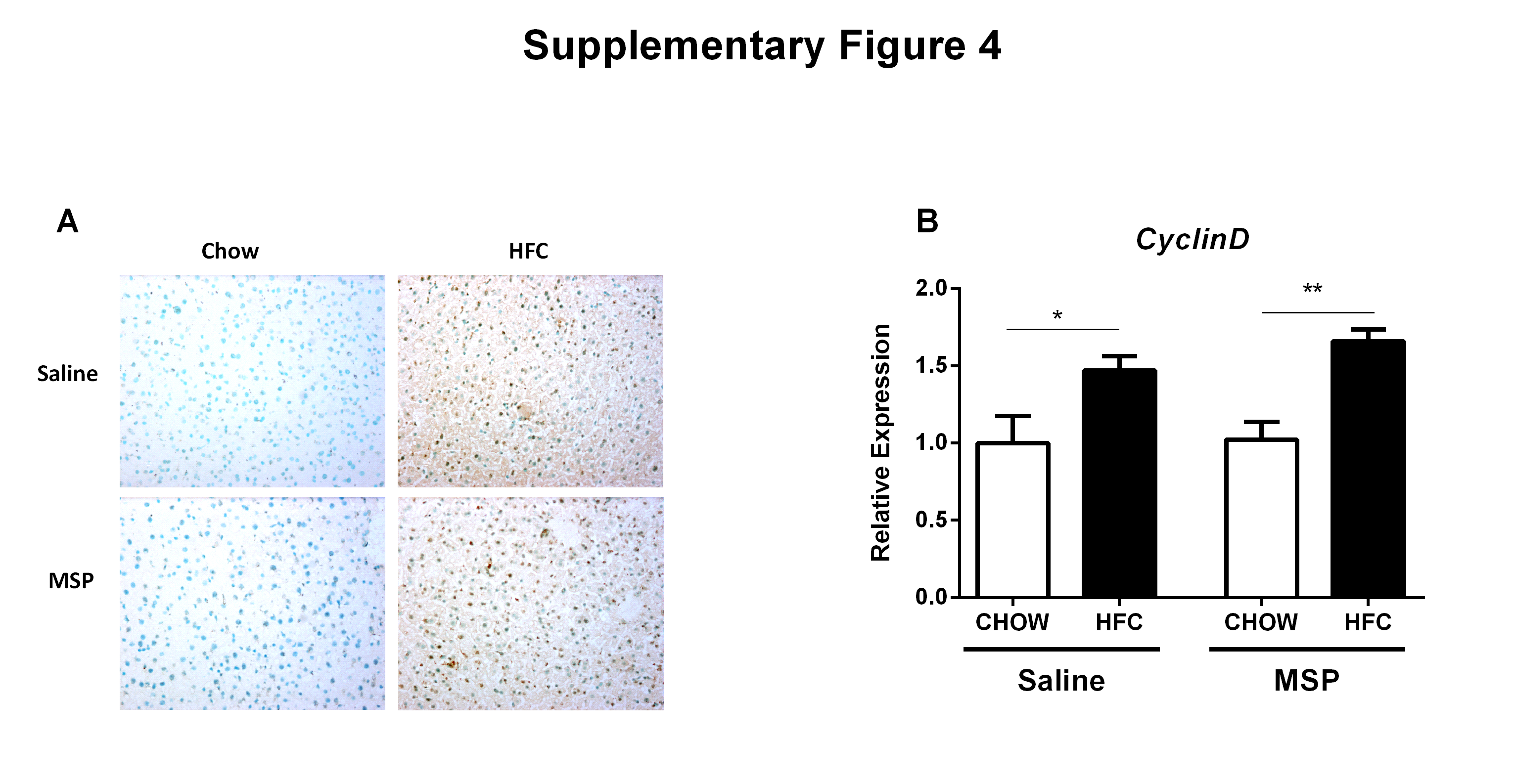

Supplement: S4 Fig — (TIF) [file pone.0163843.s004.tif]
